# ExploreModelMatrix: Interactive exploration for improved understanding of design matrices and linear models in R

**DOI:** 10.12688/f1000research.24187.2

**Published:** 2020-09-16

**Authors:** Charlotte Soneson, Federico Marini, Florian Geier, Michael I. Love, Michael B. Stadler

**Affiliations:** 1 Friedrich Miescher Institute for Biomedical Research, Basel, Switzerland; 2 SIB Swiss Institute of Bioinformatics, Basel, Switzerland; 3 Center for Thrombosis and Hemostasis, Mainz, Germany; 4 Institute of Medical Biostatistics, Epidemiology and Informatics, Mainz, Germany; 5 Department of Biomedicine, University of Basel, University Hospital Basel, Basel, Switzerland; 6 Department of Biostatistics, University of North Carolina at Chapel Hill, Chapel Hill, North Carolina, USA; 7 Department of Genetics, University of North Carolina at Chapel Hill, Chapel Hill, North Carolina, USA

**Keywords:** Linear Model, Experimental Design, Design Matrix, Shiny, R, Interactivity

## Abstract

Linear and generalized linear models are used extensively in many scientiﬁc ﬁelds, to model observed data and as the basis for hypothesis tests. The use of such models requires speciﬁcation of a design matrix, and subsequent formulation of contrasts representing scientiﬁc hypotheses of interest. Proper execution of these steps requires a thorough understanding of the meaning of the individual coefﬁcients, and is a frequent source of uncertainty for end users. Here, we present an R/Bioconductor package, *ExploreModelMatrix*, which enables interactive exploration of design matrices and linear model diagnostics. Given a sample data table and a desired design formula, the package displays how the model coefﬁcients are combined to give the ﬁtted values for each combination of predictor variables, which allows users to both extract the interpretation of each individual coefﬁcient, and formulate desired linear contrasts. In addition, the interactive interface displays informative characteristics for the regular linear model corresponding to the provided design, such as variance inﬂation factors and the pseudoinverse of the design matrix. We envision the package and the built-in collection of common types of linear model designs to be useful for teaching and self-learning purposes, as well as for assisting more experienced users in the interpretation of complex model designs.

## Introduction

Linear and generalized linear models are ubiquitous tools in a wide variety of scientific disciplines, and encompass well-known special cases such as linear and logistic regression, ANOVA and Student’s t-test. Of particular interest to us, they are also the basis for many of the most widely used tools for analysis of high-throughput biological data. This includes *limma* ^1, 2^ for linear modeling of gene expression microarray and similar data, as well as *edgeR* ^3, 4^ and *DESeq2* ^5^ for differential expression analysis of RNA-seq and other count data, *missMethyl* ^6^, *DMRcate* ^7^ and *minfi* ^8^ for differential methylation analysis, *DiffBind* ^9^ for differential binding analysis, *msmsTests* ^10^ for mass spectrometry, and many others. Since the linear model is a special case of the generalized linear model, and particularly as the aspects of defining the design matrix are shared between the two, we will generally refer to generalized linear models in the rest of this manuscript.

Fitting a generalized linear model requires observations of a response variable *y* (e.g., inferred abundance levels of a gene) as well as a set of continuous or categorical predictor variables or sample annotations (e.g. the sample genotype, age, or treatment condition). In addition, in the R statistical programming environment, the user provides a *design formula*, specifying which, and how, provided predictor variables should be used to model the expected value of the response. The design formula in R is a version of a syntax for model specification originally proposed in 1973 by Wilkinson and Rogers ^11^. This design formula and a specification of a type of contrast coding define a numeric *N × J* design matrix *X*, where *N* is the number of observations and *J* the number of model coefficients. The expected response values are then modeled by

$$E[y]=g^{-1}\left(X\beta \right),$$

where *β* = ( *β*_1_, . . . , *β _J_*) are the regression coefficients for the respective columns of the design matrix, and *g* is a link function ^12, 13^. *X β* is typically referred to as the *linear predictor*. After fitting the model, statistical tests can be performed to test the null hypothesis that a given combination of coefficients (referred to as a linear *contrast*) is zero. In this manuscript, we will focus on reference cell coding, or “treatment” coding for contrasts, though in general other schemes may also be considered. For more details on how R’s design formula functionality is implemented, we refer to the reference for statistical modeling in S ^14^.

The way that the model is specified, that is, the definition of the design matrix, naturally determines how the model coefficients should be interpreted. As an example, consider a situation with a linear model and a single categorical predictor with two levels. Defining a model including an intercept (a column of the design matrix with the value 1 for all observations) implies that the second regression coefficient represents the difference between the average response values for the two levels of the predictor, while without the intercept, the two regression coefficients directly represent the average response values for the two factor levels. Given the versatility of generalized linear models, determining the proper contrast to use for testing a specific biological hypothesis of interest requires an understanding of the interpretation of the individual regression coefficients, and can be challenging for users of generalized linear model-based tools.

Here, we present *ExploreModelMatrix* ^15^, an R package for interactive exploration of generalized linear model designs, coefficients, and contrasts. Given a table of predictor variables, the user can specify the desired design formula and explore the value of the linear predictor for each combination of predictor values, expressed in terms of the model coefficients. From this type of visualization, it is often straightforward to determine the contrast corresponding to a given comparison of interest. We envision that *ExploreModelMatrix* can be useful for both research and teaching purposes. Specifically for the latter, the application contains several built-in example data sets, corresponding to some of the most commonly used experimental design setups. The underlying function in *ExploreModelMatrix* that processes the input data and generates visualizations can also be directly called by the user, enabling the generation of static plots for inclusion in reports and educational material. It is worth noting that *ExploreModelMatrix* is not intended as a self-contained resource on generalized linear models, but rather as a complement to existing books and courses on the topic, and the application contains a list of suggested material for further study.

## Methods

### Operation

*ExploreModelMatrix* ^15^ is implemented as an R package ^16^, using the Shiny framework ^17^. The package is available via Bioconductor ^18^ (from release 3.11 onwards), with the current development version accessible via GitHub. The package has been tested with R version 3.6 and later.

An instance of the interactive application is launched by calling the ExploreModelMatrix() function. This function accepts two optional arguments; a data.frame with one row per observation and each column corresponding to a measured predictor variable (below referred to as the *sample data table*), and a design formula. If the ExploreModelMatrix() function is called without any arguments, the user can either explore one of the built-in designs, or load a sample data table from a tab-separated text file. The design formula can always be specified or modified interactively in the application. If the user wishes to generate the visualizations independently of the interactive interface, this can be achieved via the VisualizeDesign() function, which is also called internally by ExploreModelMatrix().

### Implementation

**Figure 1.**
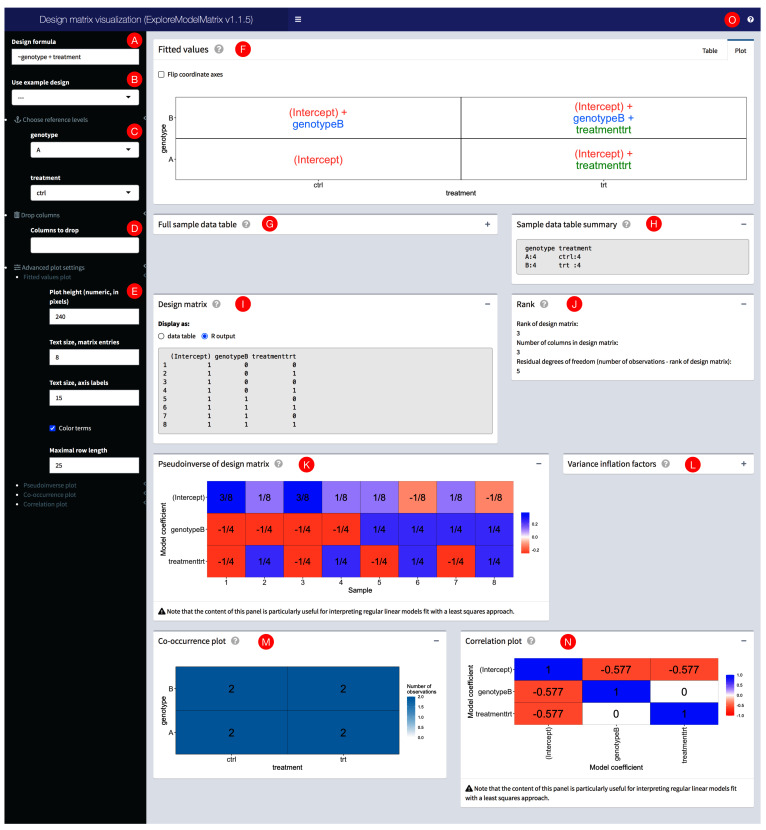
Screenshot of the *ExploreModelMatrix* interface. This example shows a model with two predictors (genotype and treatment), each with two levels, and with the assumption that their effects are additive. Red circles with letters were added to be able to refer to specific parts of the interface in the text.

The user interface of *ExploreModelMatrix* consists of a side bar with control widgets and a main window containing a set of fixed, but collapsible, panels, each illustrating a different aspect of the design matrix or the associated standard linear model ( Figure 1). A more detailed explanation of each panel is accessible via the guided tour of the interface, implemented via the *rintrojs* package ^19^ and accessible by clicking on the question mark icon in the top right corner (represented by the letter O in Figure 1). In addition, clicking on the question mark icon within a specific panel opens up the guided tour at the corresponding step.

Given a sample data table and a design formula, either provided by the user or obtained via one of the built-in designs, *ExploreModelMatrix* will first check that the two objects are compatible, i.e., that the terms in the design formula use only variables that are present in the sample data table, and that the design formula is supported by the package. If no problems are detected, *ExploreModelMatrix* will create a design matrix using the model.matrix() R function. The full sample data table, a summary of its columns, and the resulting design matrix are all displayed in the application interface for convenience (see G-I in Figure 1). In addition, the rank of the design matrix is calculated ( J). If the design matrix is not full rank, *ExploreModelMatrix* will display a warning, together with an indication of the coefficients that are not estimable (using the nonEstimable() function from the *limma* R package ^1, 2^). In addition, *ExploreModelMatrix* will inform the user if the number of rows (observations) in the design matrix is the same as its rank, in which case there are no residual degrees of freedom, and the variance or dispersion cannot be estimated.

Expressed in terms of the model coefficients, the panel in the first row of the application ( F) illustrates, in graphical and tabular form, the value of the linear predictor in a generalized linear model, for each combination of levels for the predictors used in the design formula. This provides an intuitive understanding of the interpretation of each of the model coefficients, and can be helpful for specifying appropriate contrasts.

The panels in the lower part of the interface ( K, L, N) should largely be interpreted in the context of standard linear models, where coefficient estimates are obtained using least squares fitting. The pseudoinverse *P=*( *X ^T^ X*) ^−1^ *X ^T^* ^20– 22^ represents the way each observed response value would contribute to the coefficient estimates. More precisely, in such a linear model represented by

y=Xβ+ε,

the estimated regression coefficients are given by

$$\hat{\beta }={\left(X^{T}X\right)}^{-1}X^{T}y=Py.$$

*ExploreModelMatrix* also estimates variance inflation factors and correlations among the coefficient estimates. Finally, the co-occurrence plot in the bottom left panel ( M) shows the number of observations in the data set for each combination of levels of the predictor variables.

The controls in the left-hand sidebar can be used to interactively modify the studied design as well as the display parameters of the panels. The text box in the top ( A) allows the user to type in a design formula (starting with the *~*, or “tilde” character), and the displayed figures will be updated accordingly. The dropdown menu immediately below ( B) contains the built-in example designs. To use the sample data table provided either as an argument to ExploreModelMatrix() or uploaded into the app at run time, select --- here. The next section of controls ( C) lets the user control which level should be considered the “baseline” or reference level for each categorical or factor variable in the model. *ExploreModelMatrix* will convert each character variable to a factor when a sample data table is loaded; by default the baseline level will be the first in alphabetical order.

In cases where the design matrix is not of full rank, it may be desirable to exclude a subset of the columns in the design matrix (for example, columns with all zero values or columns that are linear combinations of other columns). This can be done in the "Drop columns" section ( D). As mentioned above, in the case of a non-full rank design matrix, *ExploreModelMatrix* will indicate which coefficients are not estimable and thus candidates for being dropped. The final group of controls ( E) provide the ability to change the way the panels are displayed, e.g. by setting the height of the plot panels and changing the size and display mode of the text.

## Use case

**Figure 2.**
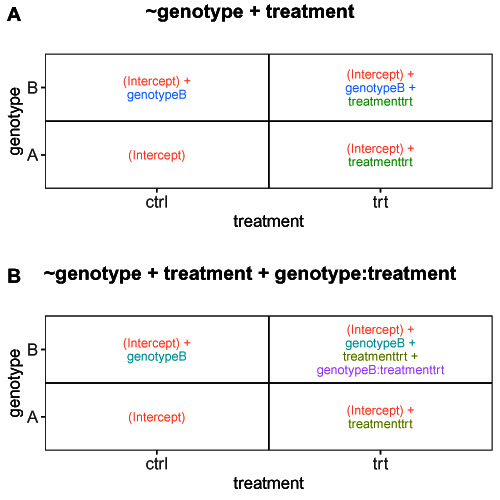
Values of the linear predictor, in terms of the model parameters, for the two-factor example design, with and without an interaction term.

Figure 1 illustrates the *ExploreModelMatrix* output for a factorial design with two predictors (genotype and treatment). We consider the effects of the two predictors to be additive, which is indicated by the design formula (~ genotype + treatment). From the graphical representation of the fitted values (also shown in Figure 2A), we can, for example, conclude that the intercept in the model directly represents the fitted value for the ‘genotype A, control’ group of samples. Similarly, the fitted value for the ‘genotype A, treated’ group is given by the sum of the intercept and the treatmenttrt coefficient. If we are interested in performing a hypothesis test to compare the treated and control groups for the samples with genotype A, we need to formulate a suitable linear contrast. Using the *ExploreModelMatrix* representation, the estimated effect size can be obtained by subtracting the fitted value for the ‘genotype A, control’ group from that of the ‘genotype A, treated’ group. The result is simply treatmenttrt, which indicates that a significance test for the difference between the two treatment groups in genotype A samples can be obtained by testing whether the coefficient treatmenttrt is zero. Interestingly, performing the same exercise in the genotype B samples yields the same result, indicating that the treatmenttrt coefficient represents the treatment effect in each of the two genotypes. This is a result of using an additive model. Changing the provided design formula to include an interaction between the two predictors (~ genotype + treatment + genotype:treatment; Figure 2B) changes the interpretation. Now, while the treatment effect in the genotype A samples is still represented by the treatmenttrt coefficient, the treatment effect in the genotype B samples is represented by the sum of the treatmenttrt and genotypeB:treatmenttrt coefficients. The interaction effect, that is, the difference between the treatment effects in the two genotype groups, is represented by the genotypeB:treatmenttrt coefficient. This example illustrates how the *ExploreModelMatrix* interface can be used to interpret coefficients in generalized linear models and create contrasts of interest.

To further illustrate how *ExploreModelMatrix* ^15^ can be used to interpret the coefficients in a complex experimental design, we consider the example of differential allele-specific expression analysis with RNA-seq data. Generalized linear models for count data often use the log link function, and we assume this to be the case in some of the interpretations below. This type of experiment contains different groups of subjects (e.g., from different experimental conditions), where each subject contributes two columns in the read count matrix: one representing the read counts for the reference allele, and one representing those for the alternative allele, for each considered gene. Typical scientific questions of interest are whether there are differences between the expression of the two alleles within each condition, and whether there are differences in the allele-specific expression patterns between the conditions. Similar setups can be observed, for example, in differential methylation experiments (where the two columns for each sample would correspond to methylated and unmethylated read counts for a feature), or in situations where individuals from different groups are each given the same set of treatments.

The sample data table considered here is provided in Table 1. In addition to the columns containing the subject identifier, the condition and the count type (reference or alternative allele), we include a column corresponding to a within-condition relabeling, or dummy encoding, of the subject identifier. Note that this dummy subject identifier has only three levels, compared to six for the original subject identifier. This design setup is available among the example designs provided within *ExploreModelMatrix*, denoted “Two crossed, one nested factor (manuscript example)”. We will illustrate two equivalent ways of setting up the design formula, and show how *ExploreModelMatrix* can help in the interpretation of the model coefficients.

**Table 1.** Sample data table for the allele-specific differential expression use case.

subject	count	condition	subjectdummy
S1	ref	control	D1
S1	alt	control	D1
S2	ref	control	D2
S2	alt	control	D2
S3	ref	control	D3
S3	alt	control	D3
S4	ref	treated	D1
S4	alt	treated	D1
S5	ref	treated	D2
S5	alt	treated	D2
S6	ref	treated	D3
S6	alt	treated	D3

First, we specify the design formula

**Figure 3.**
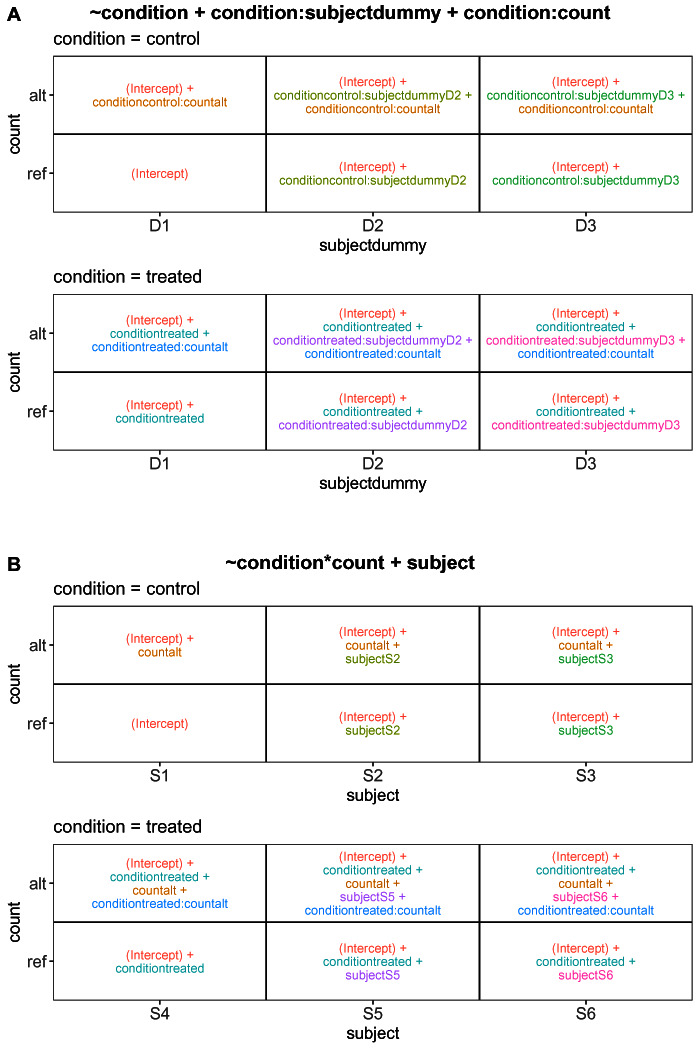
Values of the linear predictor, in terms of the model parameters, for the allele-specific expression use case. **A**. Using the design formula ~ condition + condition:subjectdummy + condition:count. **B**. Using the design formula ~ condition*count + subject.

∼condition+condition:subjectdummy+condition:count,

including an overall condition effect, a term to account for sample-specific effects, and an interaction between the condition and count type columns to capture allele-specific expression within each condition. In R’s design formula syntax, a “:” between two variable names indicates the addition of an interaction term between these two variables, which may have a different effect on columns of *X* depending on whether these are numeric or factor variables, and what other terms are in the design. Given this design formula together with the sample data table from Table 1 as the input arguments, the *ExploreModelMatrix* functions determine the composition of the linear predictor for each combination of predictor variables shown in Figure 3A (corresponds to panel ( F) in Figure 1, shown here separately for increased readability). The Rank panel in the application further indicates that the design matrix is of full rank and that the residual degrees of freedom is non-zero, allowing also estimation of variances or dispersions for use in statistical hypothesis tests involving the estimated coefficients. The illustration in Figure 3A can be used to extract appropriate contrasts for statistical testing. For example, comparing the values of the linear predictor for each sample in the control group, we can see that the conditioncontrol:countalt coefficient represents the allele-specific expression effect (alt/ref expression log-ratio) in this group. Similarly, the conditiontreated:countalt coefficient represents the allele-specific expression in the treated group. As a consequence, the condition-dependent allele-specific expression effect is obtained as the difference between the allele-specific effects within the respective conditions, that is, by conditiontreated:countalt - conditioncontrol:countalt.

Next, we illustrate an alternative way of setting up the design matrix, by specifying the design formula as

∼condition*count+subject.

Here, we use the original subject ID (not the dummy encoded), and include main effects for condition and count type as well as an interaction between the condition and the count type. In R’s design formula syntax, a “*” between two variable names indicates the addition of both main effects and an interaction term between these two variables. Upon changing the design formula in *ExploreModelMatrix*, we are notified that the design matrix is no longer full rank, as a consequence of having different subjects in the different conditions. Dropping the subjectS4 column results in a full-rank design matrix, and the composition of the linear predictor is shown in Figure 3B. The rank of the design matrix, as well as the residual degrees of freedom, are the same as with the previous formulation. However, the composition of the linear predictor for each combination of input variables is different. Comparing the alternative and reference allele groups for the control condition shows that with this formulation, the allele specificity in the control group is encoded by the countalt coefficient. Similarly, the allele specificity in the treated group is represented by the sum of the countalt and conditiontreated:countalt coefficients. Consequently, the difference in allele specificity between the treated and control group is now directly encoded in the conditiontreated:countalt coefficient.

Both model formulations can be used to analyze this type of data, and the purpose of *ExploreModelMatrix* is not to select the ‘best’ among a set of plausible models, but rather to assist the user in the interpretation of a chosen model. The example above stresses that knowing how to interpret a given coefficient in a generalized linear model is critical, that identically labelled coefficients can have different meanings depending on the chosen design formula, and that *ExploreModelMatrix* can help the user interpret the resulting coefficients for a given choice of design formula and set up an appropriate contrast.

## Summary

We have described the *ExploreModelMatrix* R/Bioconductor package ^15^, which enables interactive exploration for increased understanding of model coefficients in linear and generalized linear models. To the best of our knowledge, this is the first package of its kind, and we envision applications for both research and educational purposes. The application requires minimal input and can be launched from a local R session, as well as be deployed on a Shiny server. An example instance of the latter is available at http://shiny.imbei.uni-mainz.de:3838/ExploreModelMatrix/, and the process for deploying an instance of the application on a Shiny server is documented in one of the vignettes accompanying the software.

## Data availability

All data underlying the results are available as part of the article and no additional source data are required.

## Software availability

***ExploreModelMatrix* is available at:** http://www.bioconductor.org/packages/ExploreModelMatrix/

**Source code available at:** https://github.com/csoneson/ExploreModelMatrix.

**Source code at time of publication:** https://doi.org/10.5281/zenodo.4008415 ^15^.

**License:** MIT License.
