# Supplementary material for: ExploreModelMatrix: Interactive exploration for improved understanding of design matrices and linear models in R
Source: F1000Res. 2020 Sep 16;9:512. Originally published 2020 Jun 4. [Version 2] doi: 10.12688/f1000research.24187.2 (PMC7359746; doi:10.12688/f1000research.24187.2)
Supplement: Peer review history

# Peer review: Reviewer response for version 2

**DOI:** 10.5256/f1000research.29479.r71434

**Published:** 2020-11-02

**Authors:** Jean Fan

**Affiliations:** 1 Department of Biomedical Engineering, Johns Hopkins University, Baltimore, MD, USA; 2 Department of Computer Science, Johns Hopkins University, Baltimore, MD, USA

The authors have addressed my main concerns and the manuscript is now suitable for publication in F1000Research. 

 However, in order to ensure the utility of ExploreModelMatrix both as a guided and self-learning teaching tool, I would strongly encourage the authors to eventually conduct a user study, perhaps in a classroom setting, to assess whether their tool and the features of their tool achieve their stated goal of "helping users interpret a given design and to understand how changing the design influences the way they would perform a given comparison of interest."

 For example, I still find the "Choose reference levels" dropdown panels with the options ordered alphabetically rather by the given level ordering to be confusing from a user standpoint, particularly given that the fitted values plot does use the level ordering. While the authors suggest that the alphabetical ordering "was intentionally designed in this way" as they "reasoned that it's typically easier to find the desired level in a list of the options are listed alphabetically", which ordering is more intuitive for users is best assessed through an actual user study (rather than based on an N=1 feedback from myself).

Are the conclusions about the tool and its performance adequately supported by the findings presented in the article?

No

Is the rationale for developing the new software tool clearly explained?

Yes

Is the description of the software tool technically sound?

Yes

Are sufficient details of the code, methods and analysis (if applicable) provided to allow replication of the software development and its use by others?

Yes

Is sufficient information provided to allow interpretation of the expected output datasets and any results generated using the tool?

No

Reviewer Expertise:

Bioinformatics, Applied Statistics

I confirm that I have read this submission and believe that I have an appropriate level of expertise to confirm that it is of an acceptable scientific standard.

# Peer review: Reviewer response for version 2

**DOI:** 10.5256/f1000research.29479.r71435

**Published:** 2020-10-12

**Authors:** Lucy D'Agostino McGowan

**Affiliations:** 1 Department of Mathematics and Statistics, Wake Forest University, Winston-Salem, NC, USA

Thank you for addressing the points of my review, this paper and package will provide a nice teaching tool.

Are the conclusions about the tool and its performance adequately supported by the findings presented in the article?

Partly

Is the rationale for developing the new software tool clearly explained?

Yes

Is the description of the software tool technically sound?

Yes

Are sufficient details of the code, methods and analysis (if applicable) provided to allow replication of the software development and its use by others?

Yes

Is sufficient information provided to allow interpretation of the expected output datasets and any results generated using the tool?

Partly

Reviewer Expertise:

Biostatistics

I confirm that I have read this submission and believe that I have an appropriate level of expertise to confirm that it is of an acceptable scientific standard.

# Peer review: Reviewer response for version 1

**DOI:** 10.5256/f1000research.26680.r64395

**Published:** 2020-07-13

**Authors:** Matthew Ritchie

**Affiliations:** 1 The Walter and Eliza Hall Institute of Medical Research, Parkville, Vic, Australia

Understanding how to set up a design matrix is a significant challenge in genomic data science, especially for new analysts. The manuscript by Soneson *et al. *presents the ExploreModelMatrix R package which aims to give the user better intuition on this for any arbitrary design.

 This is great contribution and would be really useful in a teaching setting (e.g. when running an Intro to RNA-seq analysis course) to help participants understand how a linear model is parameterised and how this can be changed to suit different biological questions. At the other extreme it is also helpful in the interpretation of coefficients in more complicated designs that include interactions.

 The ExploreModelMatrix output is provided via a shiny app which allows the user to interactively change either their own design based on the data.frame supplied, or choose from a series of standard examples, which was easy to navigate. I particularly liked the interactive tour of the different elements of the interface provided by rintrojs.

 Overall, I really enjoyed using this package and list a few optional suggestions below to help further improve the work.

1. It might be useful to add a sentence about the intended audience of the package to the abstract.
2. Can a window be given to show what the line of code looks like to make the design matrix? Just thinking about beginners, who could start the app with ExploreModelMatrix(), choose an example design and then immediately see what code they would need to run at the R command prompt to create the design they're interested in (again helpful for teaching). This would provide a concrete output that the user could take forward in their analysis with a simple copy and paste. Likewise, if they provided their own data frame they will know what to do with it in terms of specifying model.matrix().
3. Would it be possible to create a dummy x vs y plot (perhaps by simulating data) to show what a fit might look like for theoretical y? This might be rather complicated to implement in practice given the wide array of models one could envisage, however, such a display would provide an intuitive view of what the coefficients represent graphically.  
4. If a model is parameterised without an intercept, it will generally be necessary to define contrasts between coefficients. Is there a way to point this out to the user in the app, or can a module be added to help set-up contrasts to show how this is done and again provide code that the analyst could then use in their analysis?
5. On page 3, column 2, paragraph 4, line 2 there appears to be a formatting problem with th'e' in 'the ExploreModelMatrix()'
6. In the 'Use cases' section, I wondered whether the simple example that features in Figure 1 could be stepped through (again to appeal to beginners) in addition to the complex ASE analysis example of Table 1 and Figure 2 which is likely to be more niche.
7. In the interactive tour of the interface, the rendering of some equations hasn't occurred properly in steps 19 and 22.
8. When you 'Flip coordinates' in the Fitted values / Co-occurrence plot, the y-axis labels aren't preserved in the flip (not sure if this is intentional)

Are the conclusions about the tool and its performance adequately supported by the findings presented in the article?

Yes

Is the rationale for developing the new software tool clearly explained?

Yes

Is the description of the software tool technically sound?

Yes

Are sufficient details of the code, methods and analysis (if applicable) provided to allow replication of the software development and its use by others?

Yes

Is sufficient information provided to allow interpretation of the expected output datasets and any results generated using the tool?

Yes

Reviewer Expertise:

Gene expression analysis

I confirm that I have read this submission and believe that I have an appropriate level of expertise to confirm that it is of an acceptable scientific standard.

# Response

**Published:** 2020-09-07

**Authors:** Charlotte Soneson

**Affiliations:** Friedrich Miescher Institute for Biomedical Research, Switzerland

Understanding how to set up a design matrix is a significant challenge in genomic data science, especially for new analysts. The manuscript by Soneson et al. presents the ExploreModelMatrix R package which aims to give the user better intuition on this for any arbitrary design. This is great contribution and would be really useful in a teaching setting (e.g. when running an Intro to RNA-seq analysis course) to help participants understand how a linear model is parameterised and how this can be changed to suit different biological questions. At the other extreme it is also helpful in the interpretation of coefficients in more complicated designs that include interactions.

 The ExploreModelMatrix output is provided via a shiny app which allows the user to interactively change either their own design based on the data.frame supplied, or choose from a series of standard examples, which was easy to navigate. I particularly liked the interactive tour of the different elements of the interface provided by rintrojs.

 Overall, I really enjoyed using this package and list a few optional suggestions below to help further improve the work.

*Thank you for your constructive comments and careful evaluation of the software. Point-by-point responses are provided below.*

 It might be useful to add a sentence about the intended audience of the package to the abstract.

*We have added a sentence about this in the abstract.*

 Can a window be given to show what the line of code looks like to make the design matrix? Just thinking about beginners, who could start the app with ExploreModelMatrix(), choose an example design and then immediately see what code they would need to run at the R command prompt to create the design they're interested in (again helpful for teaching). This would provide a concrete output that the user could take forward in their analysis with a simple copy and paste. Likewise, if they provided their own data frame they will know what to do with it in terms of specifying model.matrix().

*This is an interesting point and something that we may consider in a future version. One challenge is that different analysis packages require the design to be specified in different ways (e.g., as a design formula, or as a design matrix), and in some cases will automatically deal with e.g. non-estimable parameters. Thus, it is not obvious how to provide this code to make it as useful as possible.*

 Would it be possible to create a dummy x vs y plot (perhaps by simulating data) to show what a fit might look like for theoretical y? This might be rather complicated to implement in practice given the wide array of models one could envisage, however, such a display would provide an intuitive view of what the coefficients represent graphically.

*We agree that such plots are often instructive for the user, and some examples are included e.g. in the Data Analysis for the Life Sciences book, which we now also refer to in the application as an additional resource. Implementing them in a very general context, on the other hand, is a non-trivial task, and furthermore there is a risk that a user is misled if the coefficients estimated from the simulated response are different (perhaps with opposite signs) compared to those obtained from their actual data. For these reasons, we currently consider this out of scope for ExploreModelMatrix.*

 If a model is parameterised without an intercept, it will generally be necessary to define contrasts between coefficients. Is there a way to point this out to the user in the app, or can a module be added to help set-up contrasts to show how this is done and again provide code that the analyst could then use in their analysis?

*We have expanded on the use case section in the manuscript to provide additional guidance on how the ExploreModelMatrix interface can be used to generate contrasts.*

 On page 3, column 2, paragraph 4, line 2 there appears to be a formatting problem with th'e' in 'the ExploreModelMatrix()'

*Thanks for pointing this out. We hope that this has been resolved in typesetting of the revised version.*

 In the 'Use cases' section, I wondered whether the simple example that features in Figure 1 could be stepped through (again to appeal to beginners) in addition to the complex ASE analysis example of Table 1 and Figure 2 which is likely to be more niche.

*In the revised manuscript, we have added a paragraph walking through the design in Figure 1 and showing how ExploreModelMatrix can be used to interpret coefficients and generate contrasts of interest.*

 In the interactive tour of the interface, the rendering of some equations hasn't occurred properly in steps 19 and 22.

*This is correct - unfortunately the rintrojs package currently doesn't support proper rendering of equations via e.g. MathJax. We have initiated a discussion with the developer and hope that a solution can be engineered - in the meantime, we hope that the non-rendered equations still provide some help with the interpretation.*

 When you 'Flip coordinates' in the Fitted values / Co-occurrence plot, the y-axis labels aren't preserved in the flip (not sure if this is intentional)

*We were not able to reproduce this behaviour - when the coordinates are flipped, the labels are flipped as well. If you would be willing to open an issue in the GitHub repository with a reproducible example, that would be super helpful!*

# Peer review: Reviewer response for version 1

**DOI:** 10.5256/f1000research.26680.r64393

**Published:** 2020-07-07

**Authors:** Lucy D'Agostino McGowan

**Affiliations:** 1 Department of Mathematics and Statistics, Wake Forest University, Winston-Salem, NC, USA

**Summary**

 This article describes an R package and corresponding shiny application, ExploreModelMatrix, that can be used to explore design matrices for linear models. I can see this having a lot of utility for helping partitioners understand their design for simple research questions or for pedagogical use in the classroom. While I see this being extremely useful, the current design falls somewhere in the middle, a bit basic for complex research questions and a bit too complex for those with little statistical background. My suggestions assume that the primary user will be in the latter group.

**Major Comments**

1. The main take away seems to be from the "fitted values" boxes (both the plot and the table at the top of the application display this information). These seem to show the exact same content, so it feels repetitive to have both. The table is much more legible, but I can also see the utility of visualizing this. Perhaps these should be a *single* box with two tabs, one with the table and one with the plot. I find the content in these depictions of the fitted values confusing, the variable names here are referring to the beta coefficients from the model, not the variable values themselves. To someone familiar with R / model output this may be obvious, but I'm not sure that is the case for the target user. Additionally, the use of : to indicate an interaction is not something I would expect novice users to know. If this is meant to help users interpret/calculate fitted values after they fit their models, perhaps the "pseudo" model output could be printed above the "fitted values" plot/table. This would explain where the values that are being plugged into each of these variable names come from. Additionally, this fitted values plot becomes quickly illegible, it would be great to have some simple defaults built in to expand the plot space if there are several inputs (see #5).
2. There are several terms used in the application that may not be familiar to those without statistical/mathematical backgrounds. It would be great to have definitions provided to explain the plots/terms. For example, you could have a question mark next to each term that will pop up an explanation when the user hovers. Terms that need defining in the application: Design Matrix, Pseudoinverse of the design matrix, Variance inflation factors, Co-occurrence plot. 
3. Several of the boxes output `code` type texts (for example "Design matrix" and "Sample table summary") I am not sure what the utility of this being in this format is. If this was something the user could copy and paste into R, for example, this design choice would make sense, but I don't believe that is the purpose.
4. The first argument of the R function is `sampleData`, however everywhere else in the application/documentation this is referred to as a `sample table`. This should be consistent. 
5. There are several pieces for the user to control that could have some better defaults based on user inputs (for example the height of the plots, the size of the text, etc). Especially for a novice user, it would be great if the defaults for these values were reactive (updated based on the user input) and were *mostly* correct, allowing for tweaking only if absolutely necessary. In that case, you could hide these options in an "Advanced plot settings" tab in the sidebar, rather than having them visible when the user first opens the application. I think this would greatly improve the user experience.

**Minor Comments**

1. The "Rank" box outputs text using the textOuput() function, this means that you end up with a [1] prepending the text (which may be confusing to a novice user). Using renderUI() and uiOutput() instead would remove this [1].
2. If you remove the value from some of the numeric inputs, you end up with an error that is hard to parse ("An error has occurred. Check your logs or contact the app author for clarification."). You can check for whether the values needed for each value/plot are input using the validate() function and provide better error messages if not. (It looks like this is done for the formula input, just needs to be done for other inputs, for example, plot height, text size, etc.)

Are the conclusions about the tool and its performance adequately supported by the findings presented in the article?

Partly

Is the rationale for developing the new software tool clearly explained?

Yes

Is the description of the software tool technically sound?

Yes

Are sufficient details of the code, methods and analysis (if applicable) provided to allow replication of the software development and its use by others?

Yes

Is sufficient information provided to allow interpretation of the expected output datasets and any results generated using the tool?

Partly

Reviewer Expertise:

Biostatistics

I confirm that I have read this submission and believe that I have an appropriate level of expertise to confirm that it is of an acceptable scientific standard, however I have significant reservations, as outlined above.

# Response

**Published:** 2020-09-07

**Authors:** Charlotte Soneson

**Affiliations:** Friedrich Miescher Institute for Biomedical Research, Switzerland

Summary

 This article describes an R package and corresponding shiny application, ExploreModelMatrix, that can be used to explore design matrices for linear models. I can see this having a lot of utility for helping partitioners understand their design for simple research questions or for pedagogical use in the classroom. While I see this being extremely useful, the current design falls somewhere in the middle, a bit basic for complex research questions and a bit too complex for those with little statistical background. My suggestions assume that the primary user will be in the latter group.

*Thank you for your constructive comments. Please find point-by-point responses to each comment below.*

 Major Comments

 The main take away seems to be from the "fitted values" boxes (both the plot and the table at the top of the application display this information). These seem to show the exact same content, so it feels repetitive to have both. The table is much more legible, but I can also see the utility of visualizing this. Perhaps these should be a single box with two tabs, one with the table and one with the plot. 

*This is a good idea, and also frees up more of the horizontal screen space for the plot. In the revised version of the package (v1.1.5, available on GitHub and in the devel branch of Bioconductor), these two representations are now shown in a single box with two tabs.*

 I find the content in these depictions of the fitted values confusing, the variable names here are referring to the beta coefficients from the model, not the variable values themselves. To someone familiar with R / model output this may be obvious, but I'm not sure that is the case for the target user. Additionally, the use of : to indicate an interaction is not something I would expect novice users to know. If this is meant to help users interpret/calculate fitted values after they fit their models, perhaps the "pseudo" model output could be printed above the "fitted values" plot/table. This would explain where the values that are being plugged into each of these variable names come from. Additionally, this fitted values plot becomes quickly illegible, it would be great to have some simple defaults built in to expand the plot space if there are several inputs (see #5).

*The main motivation for this representation is to simplify the interpretation of the beta coefficients and the generation of appropriate contrasts. The actual values of the predictor variables are given as row and column names, and when combined provide a "label" for each box in the plot. For example, in the example shown in Figure 1, to find the estimated genotype effect in the control group, one would take the fitted value for the "genotype B" control samples ((Intercept) + genotypeB) and subtract the fitted value for the "genotype A" control samples ((Intercept)). The result (genotypeB) is the coefficient (column) in the design matrix to test for significance. Thus, it is essential that what is shown in the figure/table are the coefficient names (as generated by R's model.matrix()), since they are directly referred back to the column names of the design matrix. It is, in a way, less important to understand exactly why the coefficients are named as they are - the important thing is to be able to extract the proper combination of them for testing. We have addressed the automatic space allocation for the panel depending on the number of predictors and levels - see comment below.*

 There are several terms used in the application that may not be familiar to those without statistical/mathematical backgrounds. It would be great to have definitions provided to explain the plots/terms. For example, you could have a question mark next to each term that will pop up an explanation when the user hovers. Terms that need defining in the application: Design Matrix, Pseudoinverse of the design matrix, Variance inflation factors, Co-occurrence plot. 

*The tour provided with the application (accessible via the question mark icon in the top-right corner) provides some of these descriptions and additional help for interpretation. In the revised version, we have expanded on these and also added links to external documentation. In addition, we have added a question mark to each panel. Clicking on this will open the corresponding step of the built-in tour.*

 Several of the boxes output `code` type texts (for example "Design matrix" and "Sample table summary") I am not sure what the utility of this being in this format is. If this was something the user could copy and paste into R, for example, this design choice would make sense, but I don't believe that is the purpose.

*The original purpose of this formatting was to display the output as it would be shown in R, to make it easier for users to make the connection. For the sample table summary, it is also important to display the class of each column (to see, for example, if a column that displays as numbers is really a factor). In the revised version of the package, the user has the choice of whether to display the design matrix as a data table or as 'regular' R output.*

 The first argument of the R function is `sampleData`, however everywhere else in the application/documentation this is referred to as a `sample table`. This should be consistent. 

*We have updated the application to be more consistent by replacing 'sample table' by 'sample data table'. We have also updated the text in the manuscript for consistency.*

 There are several pieces for the user to control that could have some better defaults based on user inputs (for example the height of the plots, the size of the text, etc). Especially for a novice user, it would be great if the defaults for these values were reactive (updated based on the user input) and were mostly correct, allowing for tweaking only if absolutely necessary. In that case, you could hide these options in an "Advanced plot settings" tab in the sidebar, rather than having them visible when the user first opens the application. I think this would greatly improve the user experience.

*In the revised package, we have implemented a reactive panel size for the fitted values plot, which changes with the total number of displayed 'rows' (number of panels x number of levels of the predictor shown on the y-axis). It is still possible to tweak if desired. We now also collapse all plot settings in the sidebar by default.*

 Minor Comments

 The "Rank" box outputs text using the textOutput() function, this means that you end up with a [1] prepending the text (which may be confusing to a novice user). Using renderUI() and uiOutput() instead would remove this [1].

*We have changed the renderPrint() in these statements to renderText(), which removes the [1].*

 If you remove the value from some of the numeric inputs, you end up with an error that is hard to parse ("An error has occurred. Check your logs or contact the app author for clarification."). You can check for whether the values needed for each value/plot are input using the validate() function and provide better error messages if not. (It looks like this is done for the formula input, just needs to be done for other inputs, for example, plot height, text size, etc.)

*This has been fixed, and the app now displays more informative messages if numeric inputs are not properly specified.*

# Peer review: Reviewer response for version 1

**DOI:** 10.5256/f1000research.26680.r64392

**Published:** 2020-06-18

**Authors:** Jean Fan

**Affiliations:** 1 Department of Biomedical Engineering, Johns Hopkins University, Baltimore, MD, USA; 2 Department of Computer Science, Johns Hopkins University, Baltimore, MD, USA

**Executive Summary**

 Choosing between different model designs is a common first step in hypothesis testing. Here, Soneson *et al. *created an R/Bioconductor package called ExploreModelMatrix that provides an interactive Shiny interface for exploring such model designs. While the potential utility of such a package in a guided teaching setting is evident, its utility in a research setting is currently limited by its inability to accommodate larger, more complex designs common to real biological research. Most pressingly, it remains unclear how each of the explorer's modules can be used to guide a non-mathematical user to choose the appropriate model design. 

 ---

**Major comments:**

1. Overall, it is unclear to me who is intended to be the target user for this package. The authors suggest that ExploreModelMatrix can be use in biological research or teaching, where many users will not be formal mathematicians.
  However, if I try to model using redundant variables, I get a warning "The design matrix is not full rank," which is a difficult message to interpret, particularly for non-mathematicians. 
  Likewise, if the number of observations in the design matrix is the same as its rank, I get a warning "The residual degrees of freedom is 0. Values such as variances or dispersions can not be estimated from data with this design," which again is a difficult message to interpret, particularly for non-mathematicians. 
  A more actionable jargon-free set of recommendations would be important for users. 
2. In biological research, it is not uncommon to have dozens of patients and dozens of cell-types for multiple treatments across multiple time points for example. For designs with more than a few options per predictor, the current interface becomes quickly unusable. For example:
  ```
  celltype <- factor(sapply(1:10, function(x) rep(paste0('celltype', x), 30)))
  patient <- as.factor(sample(1:10, 300, replace=TRUE))
  levels(patient) <- paste0('patient', 1:10)
  names(celltype) <- names(patient) <- paste0('cell', 1:300)
  sampleData <- data.frame(patient, celltype)
  head(sampleData)
  ExploreModelMatrix(sampleData = sampleData,
                     designFormula = ~ patient + celltype)
  ```
  The 'Fitted values', 'Pseudoinverse of design matrix', and 'Correlation plot', all become overlapping and illegible, rendering the interface unusable. 
3. Testing two different design formula (one with and one without intercepts):
  ```
  ExploreModelMatrix(sampleData = sampleData,
                     designFormula = ~ genotype + treatment)
  ExploreModelMatrix(sampleData = sampleData,
                     designFormula = ~ 0 + genotype + treatment)
  ```
  I was unable to achieve the author's stated goals of "extract[ing] the interpretation of each individual coefﬁcient, and formulat[ing] desired linear contrasts" and ultimately deciding which design was best suited for my data. It is not clear how the 'variance inflation factors', 'Pseudoinverse of design matrix', 'Co-occurrence plot', and 'Correlation plot' should be used to inform my decision. 
  A video tutorial or walkthrough showing how this could be done would be useful. 

---

**Minor comments:**

1. I was unable to install the package using the instructions provided:
  ```
  > BiocManager::install("ExploreModelMatrix")
  Bioconductor version 3.9 (BiocManager 1.30.10), R 3.6.0 (2019-04-26)
  Installing package(s) 'ExploreModelMatrix'
  Warning message:
  package ‘ExploreModelMatrix’ is not available (for R version 3.6.0) 
  ```
  Instead, I had to use:
  ```
  devtools::install_github('csoneson/ExploreModelMatrix')
  ```
2. The "Choose reference levels" dropdown panels order the options alphabetically rather by the given level ordering. The fitted values plot does use the correct order though. 
3. All the functions and utilities that are available are not clear. For example, the "Flip coordinates" toggle was very useful and it took me awhile to find it. Again, video tutorial or walkthrough highlighting these features will be very helpful.
4. If the annotation names ("subjectdummy", "condition", "count", etc) is too long, the Fitted values visualization becomes illegible as the text overlaps each other without wrapping. 

Are the conclusions about the tool and its performance adequately supported by the findings presented in the article?

No

Is the rationale for developing the new software tool clearly explained?

Yes

Is the description of the software tool technically sound?

Yes

Are sufficient details of the code, methods and analysis (if applicable) provided to allow replication of the software development and its use by others?

Yes

Is sufficient information provided to allow interpretation of the expected output datasets and any results generated using the tool?

No

Reviewer Expertise:

Bioinformatics, Applied Statistics

I confirm that I have read this submission and believe that I have an appropriate level of expertise to confirm that it is of an acceptable scientific standard, however I have significant reservations, as outlined above.

# Response

**Published:** 2020-09-07

**Authors:** Charlotte Soneson

**Affiliations:** Friedrich Miescher Institute for Biomedical Research, Switzerland

Executive Summary

Choosing between different model designs is a common first step in hypothesis testing. Here, Soneson et al. created an R/Bioconductor package called ExploreModelMatrix that provides an interactive Shiny interface for exploring such model designs. While the potential utility of such a package in a guided teaching setting is evident, its utility in a research setting is currently limited by its inability to accommodate larger, more complex designs common to real biological research. Most pressingly, it remains unclear how each of the explorer's modules can be used to guide a non-mathematical user to choose the appropriate model design. 

*Thank you for your constructive comments. We provide point-by-point responses below. Generally, we would like to emphasize that the main purpose of the application is to help users interpret a given design, and to understand how changing the design influences the way you would perform a given comparison of interest, rather than choosing a design for a specific experiment (the latter needs to be guided by insights and expectations about the system at hand, and possibly in consultation with a statistical collaborator).*

---

Major comments:

Overall, it is unclear to me who is intended to be the target user for this package. The authors suggest that ExploreModelMatrix can be use in biological research or teaching, where many users will not be formal mathematicians.

However, if I try to model using redundant variables, I get a warning "The design matrix is not full rank," which is a difficult message to interpret, particularly for non-mathematicians. 

Likewise, if the number of observations in the design matrix is the same as its rank, I get a warning "The residual degrees of freedom is 0. Values such as variances or dispersions can not be estimated from data with this design," which again is a difficult message to interpret, particularly for non-mathematicians. 

A more actionable jargon-free set of recommendations would be important for users. 

*In the revised version, we have expanded on these messages and added links to external documentation. We have also expanded the built-in tour, which provides additional interpretation assistance for the different concepts.*

In biological research, it is not uncommon to have dozens of patients and dozens of cell-types for multiple treatments across multiple time points for example. For designs with more than a few options per predictor, the current interface becomes quickly unusable. For example:

```

celltype <- factor(sapply(1:10, function(x) rep(paste0('celltype', x), 30)))

patient <- as.factor(sample(1:10, 300, replace=TRUE))

levels(patient) <- paste0('patient', 1:10)

names(celltype) <- names(patient) <- paste0('cell', 1:300)

sampleData <- data.frame(patient, celltype)

head(sampleData)

ExploreModelMatrix(sampleData = sampleData,

                   designFormula = ~ patient + celltype)

```

The 'Fitted values', 'Pseudoinverse of design matrix', and 'Correlation plot', all become overlapping and illegible, rendering the interface unusable. 

*It is true that with a large number of levels for each factor, the available screen space can be too small. There are several ways around this. First, the size of the displayed text as well as of the panels can be modified. In the revised package we have implemented a reactive panel height for the fitted values plot, which should avoid the need for the user to change it manually. Furthermore, by combining the plot and table representations of the fitted values in one panel, the full width of the application is used for the plot. Second, if the number of levels becomes very large, it may be better to use the non-interactive interface where the output can be written to a file of arbitrary dimensions. Third, in order to understand a given type of model, it is often possible to work with a reduced version, with fewer levels per predictor but the same underlying structure.*

Testing two different design formula (one with and one without intercepts):

```

ExploreModelMatrix(sampleData = sampleData,

                   designFormula = ~ genotype + treatment)

ExploreModelMatrix(sampleData = sampleData,

                   designFormula = ~ 0 + genotype + treatment)

```

I was unable to achieve the author's stated goals of "extract[ing] the interpretation of each individual coefﬁcient, and formulat[ing] desired linear contrasts" and ultimately deciding which design was best suited for my data. It is not clear how the 'variance inflation factors', 'Pseudoinverse of design matrix', 'Co-occurrence plot', and 'Correlation plot' should be used to inform my decision. 

A video tutorial or walkthrough showing how this could be done would be useful. 

*The main purpose of the application is to help users interpret a given design, and to understand how changing the design influences the way you would perform a given comparison of interest, rather than choosing a design for a specific experiment (the latter needs to be guided by insights and expectations about the system at hand). In the example above, the fitted values panel will inform the user about, e.g., the contrast required to compare two of the treatment groups. Neither design is more 'suitable' for the data, but depending on which one is selected, the contrast needs to be adapted.*

---

Minor comments:

I was unable to install the package using the instructions provided:

```

> BiocManager::install("ExploreModelMatrix")

Bioconductor version 3.9 (BiocManager 1.30.10), R 3.6.0 (2019-04-26)

Installing package(s) 'ExploreModelMatrix'

Warning message:

package ‘ExploreModelMatrix’ is not available (for R version 3.6.0) 

```

Instead, I had to use:

```

devtools::install_github('csoneson/ExploreModelMatrix')

```

*ExploreModelMatrix was added to Bioconductor in release 3.11 (the current release, since April 2020). Thus, in order to install it via BiocManager::install() you need to have the most recent Bioconductor release. We now mention this in the manuscript, and we have expanded a bit on the installation instructions in the README to clarify this point.*

The "Choose reference levels" dropdown panels order the options alphabetically rather by the given level ordering. The fitted values plot does use the correct order though. 

*This was intentionally designed in this way - if the user wants to choose a new reference level, the current level ordering doesn't matter, and we reasoned that it's typically easier to find the desired level in a list if the options are listed alphabetically.*

All the functions and utilities that are available are not clear. For example, the "Flip coordinates" toggle was very useful and it took me awhile to find it. Again, video tutorial or walkthrough highlighting these features will be very helpful.

*The application contains a walkthrough (accessible via the question mark in the top-right corner), pointing out useful features. Moreover, we have added a question mark button to each panel, which will open up the corresponding step in the tour. We have also moved the "Flip coordinates" toggle inside the panel with the fitted values plot for easier access.*

If the annotation names ("subjectdummy", "condition", "count", etc) is too long, the Fitted values visualization becomes illegible as the text overlaps each other without wrapping. 

*This is difficult to manage automatically, since the actual width of the panel depends on the size of the browser window (as opposed to the height, which is set in pixels). It is best remedied by changing the font size of the displayed text in the side bar. We have intentionally kept the full coefficient name on a single line, to avoid misinterpretations and make it easier to match it back to the column names of the design matrix.*
